# Principal component analysis for predicting transcription-factor binding motifs from array-derived data

**DOI:** 10.1186/1471-2105-6-276

**Published:** 2005-11-18

**Authors:** Yunlong Liu, Matthew P Vincenti, Hiroki Yokota

**Affiliations:** 1 Department of Biomedical Engineering, Indiana University – Purdue University Indianapolis, Indianapolis, IN 46202, USA.; 2 Weldon School of Biomedical Engineering, Purdue University, West Lafayette, IN 47907, USA.; 3 Department of Anatomy and Cell Biology, Indiana University – Purdue University Indianapolis, Indianapolis, IN 46202, USA.; 4 Department of Veteran's Affairs, White River Jct, VT 05009, USA.; 5 Department of Medicine, Dartmouth Medical School, Hanover, NH 03755, USA.

## Abstract

**Background:**

The responses to interleukin 1 (IL-1) in human chondrocytes constitute a complex regulatory mechanism, where multiple transcription factors interact combinatorially to transcription-factor binding motifs (TFBMs). In order to select a critical set of TFBMs from genomic DNA information and an array-derived data, an efficient algorithm to solve a combinatorial optimization problem is required. Although computational approaches based on evolutionary algorithms are commonly employed, an analytical algorithm would be useful to predict TFBMs at nearly no computational cost and evaluate varying modelling conditions. Singular value decomposition (SVD) is a powerful method to derive primary components of a given matrix. Applying SVD to a promoter matrix defined from regulatory DNA sequences, we derived a novel method to predict the critical set of TFBMs.

**Results:**

The promoter matrix was defined to establish a quantitative relationship between the IL-1-driven mRNA alteration and genomic DNA sequences of the IL-1 responsive genes. The matrix was decomposed with SVD, and the effects of 8 potential TFBMs (5'-CAGGC-3', 5'-CGCCC-3', 5'-CCGCC-3', 5'-ATGGG-3', 5'-GGGAA-3', 5'-CGTCC-3', 5'-AAAGG-3', and 5'-ACCCA-3') were predicted from a pool of 512 random DNA sequences. The prediction included matches to the core binding motifs of biologically known TFBMs such as AP2, SP1, EGR1, KROX, GC-BOX, ABI4, ETF, E2F, SRF, STAT, IK-1, PPARγ, STAF, ROAZ, and NFκB, and their significance was evaluated numerically using Monte Carlo simulation and genetic algorithm.

**Conclusion:**

The described SVD-based prediction is an analytical method to provide a set of potential TFBMs involved in transcriptional regulation. The results would be useful to evaluate analytically a contribution of individual DNA sequences.

## Background

The use of microarrays has led to a significant number of exciting discoveries establishing important links between mRNA expression patterns and cellular states [1,2]. Mathematical and computational models have been developed to understand and characterize the molecular mechanisms underlying expression patterns [3,4]. However, it remains difficult to discover and validate novel transcription-factor binding motifs (TFBMs) in the human genome. The popular approach to identify TFBMs utilizes sequence comparisons among co-expressed genes [5] or across multi-species [6]. Although any consensus motif can be searched among the co-regulated genes in hierarchical clusters [7,8], this approach is not aimed to build a global model with multiple binding motifs. TFBM can be inspected through phylogenetic footprinting [6,9,10], but identifying orthologous genes and their associated regulatory regions are not always possible. Model-based approaches, initially developed using yeast genome [3], encounter difficulty in evaluating the astronomical number of TFBM selections in the combinatorial problem [11,12]. Although multiple binding motifs were selected in the yeast dataset using a recursive formula, prediction of TFBMs would be affected depending on the order of selected motifs [3]. Some models lack statistical standards for determining the number of TFBMs having combinatorial roles that are critical in expression patterns. Thus, a predictive model that provides a comprehensive set of TFBMs still needs to be developed.

The specific aim of the current study was to devise a model for predicting known and *de novo *transcription factor binding motifs from array-derived mRNA expression levels by developing a unique principal component analysis. We employed the responses of human chondrocytes to interleukin-1 (IL-1) as a model system [13]. IL-1 is a pro-inflammatory cytokine, and it stimulates not only inflammatory responses but also tissue degeneration [5]. More than 100 microarray analyses have been conducted to analyze IL-1-driven responses in various cell types, including chondrocytes [14,15], and significant efforts have been made to understand transcriptional mechanisms of IL-1 response [16-18]. However, few of the previous studies have validated the global roles of multiple critical TFBMs in downregulation or upregulation of a cluster of genes.

**Figure 1:**
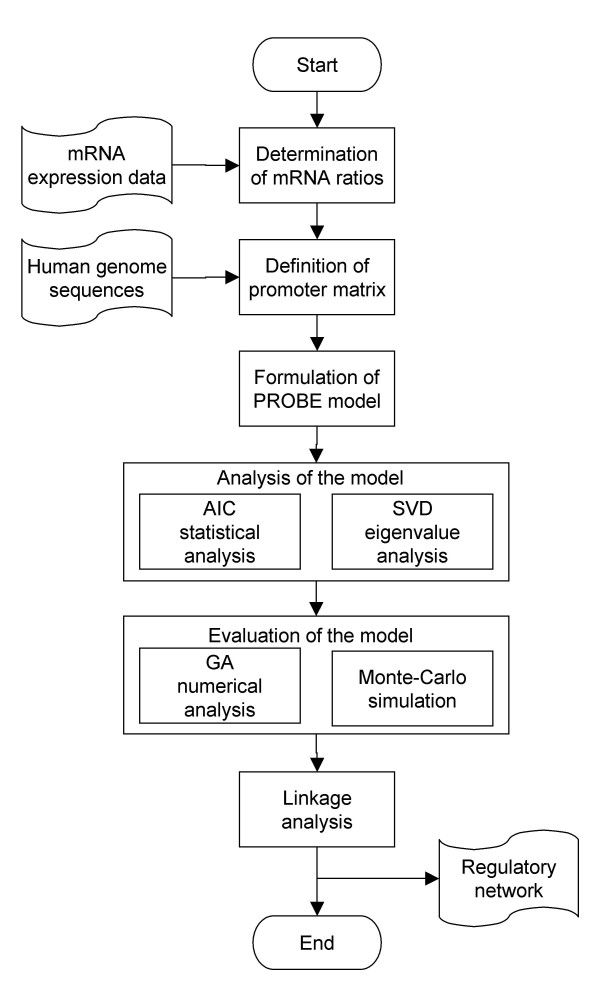
**Flowchart of the model-based analysis of transcription factor binding motifs (TFBMs)**. The mRNA expression data and the human genome sequence information were used to formulate the mathematical model. The putative TFBMs were selected through the Akaike Information Criterion (AIC) analysis and the Singular Value Decomposition (SVD) eigen value analysis. The predicted TFBMs were evaluated with the genetic algorithm (GA) numerical analysis and the Monte-Carlo simulation, and the model-based TFBM network was linked to the known transcription factors and their binding motifs.

In this principal component analysis, we introduced the Akaike information criterion (AIC) test, singular value decomposition (SVD), and a genetic algorithm (GA) to predict and evaluate TFBMs from a pool of random DNA sequences (Fig. 1). The predictive model was formulated using state vectors, which represented a contribution of potential TFBMs to the IL-1 responses. The promoter matrix was defined to build the quantitative relationship between the mRNA expression vector and the state vectors, and a unique SVD procedure was applied to the promoter matrix. Although one previous study defined the mRNA expression level as a state variable, dynamical correlations among the mRNA levels do not directly represent biological processes [19]. Here, a state variable was defined as an activation level of each TFBM, and SVD was used to link the primary components in the expression vector to the influential TFBM candidates in the state vector through the eigen gene vectors and the eigen TFBM vectors. The analytical prediction of TFBMs with SVD was evaluated numerically using Monte Carlo simulation and GA.

## Results

Prediction and validation of novel and known TFBMs were conducted using logarithmic ratios of the IL-1-driven mRNA alterations in human chondrocytes (Fig. 1). First, AIC was used to determine a statistically meaningful number of TFBMs in the model. Second, the contribution of each of the 512 TFBM candidates to the IL-1 responses was evaluated by decomposing the promoter matrix with SVD. Third, the SVD-based priority of TFBMs was evaluated numerically by GA and Monte-Carlo simulation. Fourth, a linkage was established among the predicted and known TFBMs.

### Messenger RNA ratios and AIC analysis

**Figure 2:**
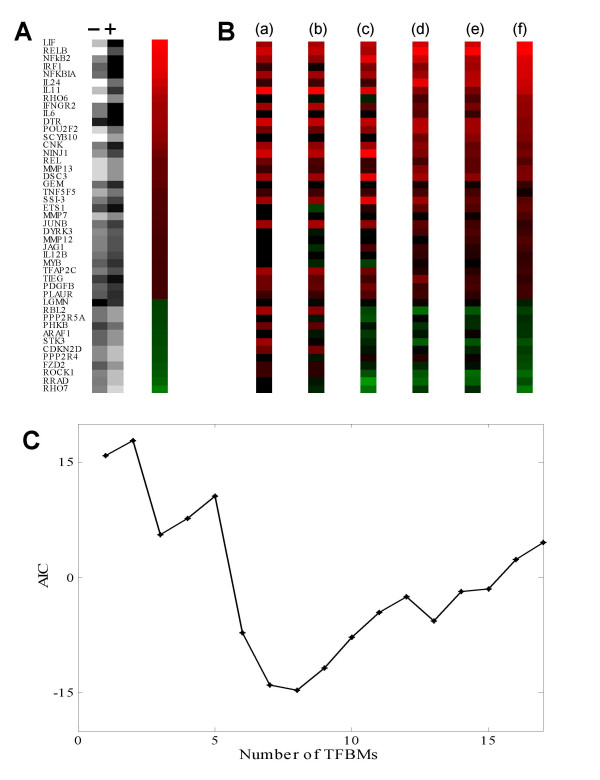
**Selection of 45 IL-1-responsive genes and AIC analysis**. (A) Ratios of mRNA expression in chondrocytes. The grayscale columns marked "-" and "+" represent the mRNA levels without and with the IL-1 treatment, respectively. The color-coded column displays the logarithmic mRNA expression ratio (the mRNA level in cells treated with IL-1 to the untreated control level). The darker color indicates the greater alteration, and "red" and "green" illustrate up- and down-regulation, respectively. (B) Modeled mRNA ratios based on the 300-bp upstream regulatory DNA region. As TFBM candidates, 512 DNA fragments, 5 bp in length, were considered. The mathematical models with (a) 1, (b) 2, (c) 4, (d) 8, (e) 16, and (f) 32 putative TFBMs are illustrated. (C) AIC analysis. The minimum AIC value was obtained when the number of TFBMs was 8.

Using data obtained in primary cultures of human articular chondrocytes, 45 IL-1-responsive genes were selected and the ratios of mRNA levels from IL-1-treated cells against mRNA levels in untreated cells were calculated from the list of IL-1-responsive genes in primary chondrocytes published by Vincenti and Brinckerhoff [13]. As shown in Fig. 2A, the relative mRNA levels are represented in a greyscale, and the logarithmic ratios are illustrated in a green to red color code. The mRNA ratios for 33 genes were positive (upregulation; indicated by green), while the ratios for 12 genes were negative (downregulation; indicated by red). Using Eq. (1) and the SVD procedure, these logarithmic mRNA ratios were modelled against 1 to 32 TFBMs that were chosen from random DNA sequences of 5 bp in length (Fig. 2B). As expected, the model error decreased monotonically as the number of TFBMs increased from 1 to 32. In order to estimate the proper number of TFBMs in the model, AIC was calculated using Eq. (2) (Fig. 2C). The minimum AIC was obtained with 8 TFBMs, which were used as models for further analysis.

### SVD analysis

**Figure 3:**
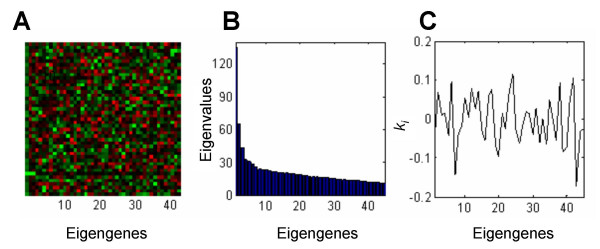
**SVD analysis for the 45 IL-1-responsive genes**. (A) Forty-five eigen genes in the matrix *U *in *H *= *UΛV*^*T*^. (B) Eigen values, *λ*_1_, *λ*_2_,..., *λ*_45_, in the matrix *Λ*. (C) Weighting factors, *k*_*i*_, for the *i-th *eigen gene.

**Figure 4:**
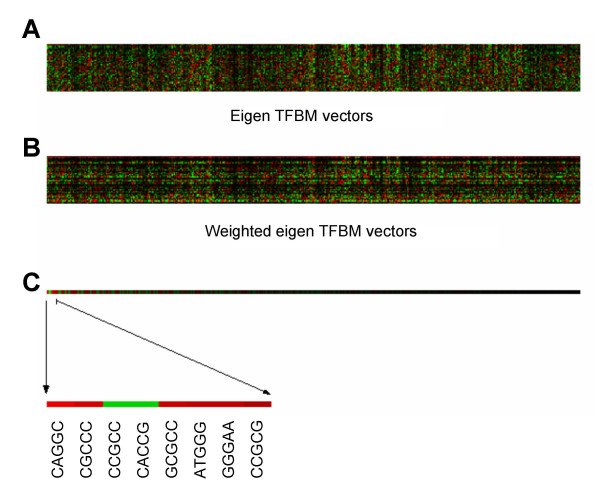
**SVD-based selection of TFBMs**. (A) Eigen TFBM vectors in the matrix *V*^*T *^in *H *= *UΛV*^*T*^. (B) Weighted eigen TFBM vectors with the weighting factor, *k*_*i*_. (C) Putative TFBMs predicted from the SVD analysis.

Using the SVD procedure, the promoter matrix H, built from the 300-bp upstream flanking sequences, was factorized into three matrices in Eq. (4). Using the eigen gene vectors in U (Fig. 3A) and the eigen values in Λ (Fig. 3B), the observed mRNA ratios were decomposed linearly with definition of the weighing factors, *k*_*i *_(Fig. 3C), in Eq. (5). Out of 45 eigen values, the primary and the secondary eigen values were 133.4 and 64.6. Shannon entropy was calculated as 0.65 [6], and the eigen values suggested a relatively even spread distribution among the 45 eigen gene vectors. Note that that Shannon entropy takes values between 0 and 1, and a smaller value suggests that expression data are dominated by influential eigen values. Using the weighing factors for each of the eigen TFBM vectors, the most influential 8 TFBMs, whose contribution to the expression levels of IL-1-responsive genes was predicted to be larger than the others, were selected. First, the eigen TFBM vectors (Fig. 4A) were derived as a complement of the eigen gene vectors. Then, each TFBM candidate in the eigen TFBM vectors was weighted by the same weighting factors defined in Eq. (5). This weighting process predicted the contributions of TFBM candidates to the observed value of *z* (Fig. 4B). Lastly, the overall significance to the selected 45 genes was estimated by adding the 45 row elements in the eigen TFBM vectors (Fig. 4C). The predicted TFBM candidates were 5'-CAGGC-3', 5'-CGCCC-3', 5'-CCGCC-3', 5'-CACCG-3', 5'-GCGCC-3', 5'-ATGGG-3', 5'-GGGAA-3', and 5'-CCGCG-3'.

### GA analysis, Monte-Carlo simulation, and leave-one-out test

**Figure 5:**
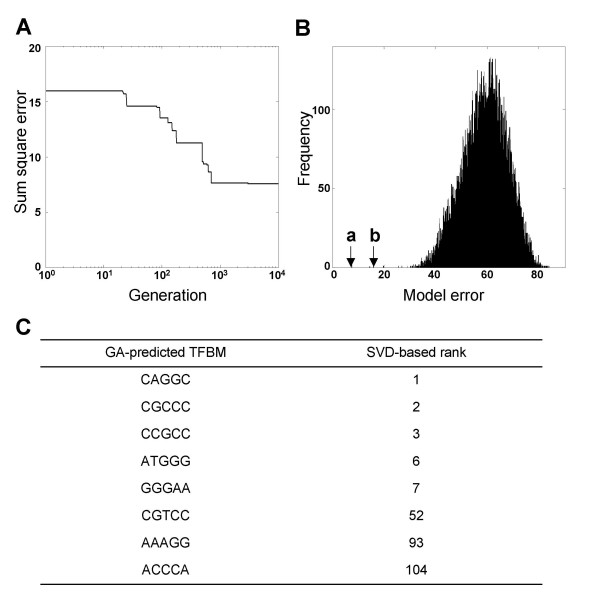
**GA analysis and Monte-Carlo simulation**. (A) Evolution of the model error in the GA analysis during 10,000 generations. (B) Model error in Monte-Carlo simulation. The labels, *a *and *b*, indicate the error in the GA analysis and the SVD analysis, respectively. (C) Comparison between the GA-predicted TFBMs and the SVD-predicted TFBMs.

In order to evaluate the selection of 8 TFBMs based on the above principal component analysis, the numerical search for TFBM candidates was conducted with the GA analysis. Starting with 200 digital chromosomes in Eq. (6), including the chromosome for the SVD solution, the population of chromosomes was evolved for 10^4 ^generations. During evolution, the model error was reduced through artificial chromosome recombinations and mutations (Fig. 5A). The sum square error for the mRNA ratios was 15.94 (SVD solution) and 7.55 (GA solution). These values were smaller than the Monte-Carlo results of 58.97 ± 8.61 (N = 10,000) using a random selection of TFBMs (Fig. 5B). The GA solution reduced the error of the SVD solution by 52.6% by retaining five SVD-driven TFBMs and introducing three new TFBMs, 5'-CGTCC-3', 5'-AAAGG-3', and 5'-ACCCA-3' (Fig. 5C).

**Figure 6:**
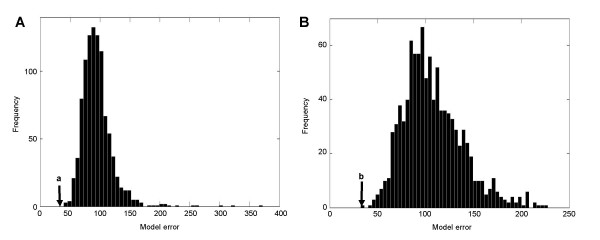
**Leave-one-out test**. (A) Model error with a randomized promoter matrix. The mean and s.d. of sum square error is 108 ± 31 (N = 1,000), and the label "*a*" indicates the error with the original promoter sequences. (B) Model error with randomized gene expression ratios. The mean and s.d. of sum square error is 93 ± 23 (N = 1,000), and the label "*b*" indicates the error with the original gene expression ratios.

In order to further examine the SVD-based model, we conducted a leave-one-out test. In this test, (N - 1) genes were used to build a model and one gene was used to validate the model through any difference between the observed and the predicted expression levels. The process was repeated N times (N = 45) by removing one gene at a time. The model error for a complete set of leave-one-out tests was 33. To evaluate significance of the leave-one-out model error, Monte-Carlo analyses were conducted using two datasets. In the first dataset the elements in the promoter matrix was reshuffled, and in the second dataset the order of mRNA expression levels was randomized. The model error was 108 ± 31 (mean ± s.d.) and 93 ± 23 for the first and the second datasets, respectively (Fig. 6).

### Linkage to known TFBMs

**Figure 7:**
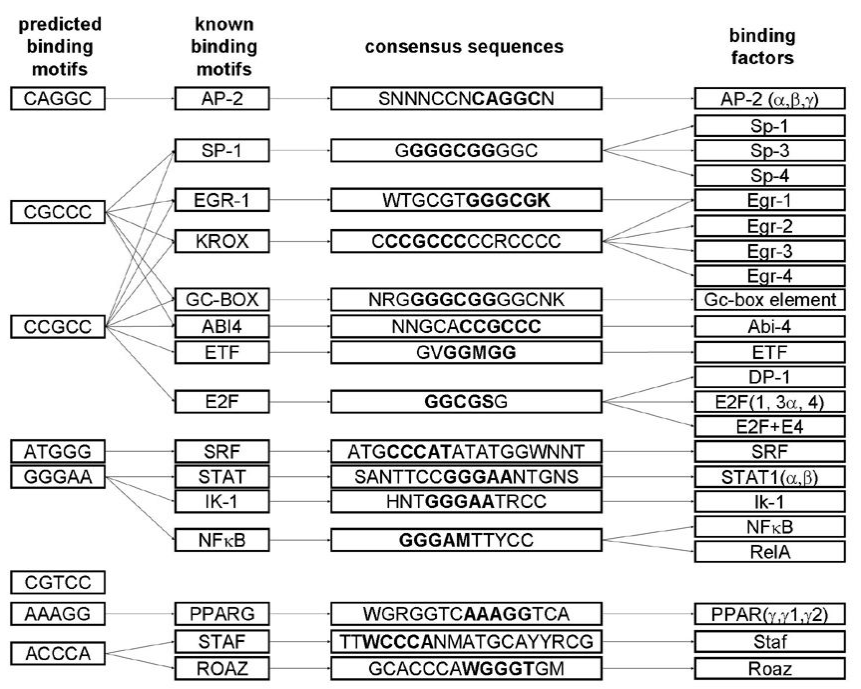
**Linkage between the predicted TFBMs and the biologically known TFBMs**. Eight TFBMs, derived from the GA analysis, were linked to the known biological motifs with the list of consensus sequences. The abbreviations are R (A, G), Y (C, T), K (G, T), W (A, T), S (C, G), B (C, G, T), D (A, G, T), H (A, C, T), V (A, C, G), and N (A, C, G, T). The binding factors (transcription factors) to the consensus sequences are included.

The 8 TFBM candidates obtained from the GA analysis were graphically linked to the known TFBMs (Fig. 7). The GA-based TFBMs are represented by 8 boxes in the first column, and each box is linked to the biologically known TFBMs such as AP2, SP1, EGR1, etc. For instance, 5'-CGCCC-3', one of the TFBMs predicted by GA, is part of consensus sequences of SP1, EGR1, KROX, GC-BOX, and ABI4.

## Discussion

In this report, we have presented a predictive model and its validation using the transcriptional responses to IL-1 in human chondrocytes as a model system. From a pool of 512 random DNA sequences of 5 bp in length as potential TFBM candidates, the SVD analysis and the GA simulation both identified 8 TFBMs. Five out of 8 TFBM candidates were identical in both analyses, and several of the known TFBMs, including AP2, EGR1, GC-BOX, SP1, NFκB, and LEF1, coincided with the predicted TFBMs.

Prior to application to the mammalian gene expression in the current study, the described approach was examined to build a model for a Ras/cAMP signal transduction pathway in yeast. This pathway is well characterized in yeast, and a cAMP responsive element (CRE; 5'-[A/G][A/C][T/C]GCAGT-3'), which is conserved in eukaryotes, is known to be involved. The SVD-based approach with 5-bp sequences predicted a part of CRE (5'-AATGC-3') together with two yeast-specific binding motifs such as 5'-AGGGG-3' (binding motif for MSN2/MSN4; stress responsive element) and 5'-ACCGG-3' (binding motif for LEU3). Since both MSN2 and LEU3 are differentially expressed in response to Ras activation [5], the results allowed us to apply this principal component approach to the current study on the human IL-1 responses (see additional file).

In the prediction phase of TFBM analysis, we demonstrated that the SVD analysis prioritized the contribution of individual TFBM candidates, and the GA algorithm was employed to evaluate independently the SVD solution. SVD is computationally inexpensive, and the results are reproducible since no random parameters are involved. It is straightforward to incorporate the effects of degenerate binding sequences by modifying a linear combination of the eigen TFBM vectors and adding contributions from redundant sequences in the final SVD procedure. More specifically, to any TFBM candidate there are 15 degenerate motifs with one base-pair mismatch and the contribution of these degenerate sequences can be included in the model with an appropriate weighting factor. The standard computational complexity of SVD procedure is estimated as O(*m*^2^*n*) or O(*mn*^2^) [20]. The complexity can be reduced to O(*mn*) by implementing the average algorithm or employing parallel computing [21]. GA is a heuristic solver suitable for searching efficiently the suboptimal solutions. There are 1.1 × 10^17 ^combinations to predict 8 TFBMs from 512 candidates in this study. It is virtually impossible to evaluate all combinations, although either SVD- or GA-based TFBM prediction is not globally optimal in terms of minimization of the prediction error. A predicted 5-bp TFBM can represent more than one motif longer than 5 bp sequences.

The use of mathematical and computational procedures such as AIC, SVD, and GA have been used previously to analyze the behaviour of complex biological systems [22,23]. In prediction of TFBMs from the microarray data, however, the described usage here is unique in a novel state-variable representation. Since many genes are regulated by multiple TFBMs, a statistical standard such as AIC may be used appropriately to validate the number of TFBMs that are meaningful in array-derived data. The previous use of SVD has been limited to clustering expression patterns in the eigen gene space [22,24]. The unique feature of the described predictive model is to link the eigen gene space to the eigen TFBM space by applying SVD to the promoter matrix defined from TFBMs. Evolutionary algorithm such as GA has been used to estimate the values of parameters [25,26]. We employed GA to select the set of TFBMs from an artificial chromosome that is composed of on/off switches for 512 random DNA sequences.

The predictive model in this study generated many testable hypotheses on known TFBMs, as well as novel TFBM candidates, and led us to the analysis of transcription factors. Five out of the 8 TFBM candidates were linked to known transcription factors. Among them, AP2 is known to be involved in stress responses [27] and LEF1 is known to be involved in a wnt signalling pathway [28]. However, neither AP2 nor LEF1 is reported to be responsive to IL-1. EGR1 increases expression of inflammatory cytokines and is involved in IL-1-induced downregulation of the type II collagen promoter in chondrocytes [29], and the GC-box is a widely distributed promoter component. The binding site of SP1 is recognized by SP3, which may oppose positive effects of SP1 [30]. NFκB is a pivotal transcription factor that is both induced at the mRNA level, as shown here, and activated by proinflammatory cytokines [31-33]. However, the relatively long degenerate consensus sequence of its binding site 5'-GGG(A/G)(C/A/T)T(T/C)(T/C)CC-3'requires a further linkage analysis to the predicted TFBM of 5'-GGGAA-3'. In a separate study, the promoter competition assay was conducted to evaluate the role of the SVD-selected TFBMs using three IL-1-responsive genes, LIF, NFκB2, and IRF1 [34]. In the assay, the stimulatory effects of 5'-CAGGC-3' and 5'-CGCCC-3', as well as the inhibitory effects of 5'-CCGCC-3', 5'-CACCG-3', and 5'-GCGCC-3', were consistent to the SVD prediction. In order to further validate the stimulatory role of 5'-CAGGC-3', a gel shift assay was conducted. As predicted, incubation with the nuclear extracts isolated from the IL-1-treated cells retarded the mobility of the DNA fragment containing 5'-CAGGC-3' (see additional file).

The described state-variable formulation of the predictive model can be extended to include redundancy in TFBM consensus sequences, temporal mRNA profiles, and interactions of TFBMs with transcription factors and cofactors. Short motifs such as 5 bp TFBMs in this study may present less specificity, but the described SVD procedure can increase specificity easier than any combinatorial search algorithm such as GA. The model can be extended to predict a dynamical state of TFBMs associated with the regulation of the temporal mRNA expression profiles [23]. Interactions among TFBMs through transcription factors and cofactors can be implemented through the nonlinear version [35].

## Conclusion

Identification of TFBMs in the human genome is critically important in the post Human Genome Project era [36]. Although experimental evaluation is mandatory to gain biological insights from the model-based predictive results, an analytical model at nearly no computational cost would be useful to provide initial conditions for numerical optimization or predict a set of potential targets for experimental verification. Although the prediction is dependent on definition of regulatory regions, the described model-based analysis allowed us to gain a new biochemical insight on the IL-1 responses by integrating the SVD procedure and Akaike information criterion. In conclusion, the current study on gene responses to IL-1 demonstrates that application of the primary component analysis would predict and validate the novel and known TFBMs from the microarray data using genomic DNA information.

## Methods

### Determination of mRNA ratios

The mRNA expression data for the IL-1-responsive genes in primary cultures of human articular chondrocytes were obtained from the lists published by Vincenti and Brinckerhoff [13]. The logarithmic ratios of mRNA levels in IL-1β (10 ng/ml)-treated chondrocytes to control mRNA levels were determined for 45 IL-1-responsive genes, whose transcription initiation site was identifiable in the GenBank sequences or by the PEG program [37,38]. The logarithmic ratio makes it easy to characterize both upregulation and downregulation to the control level, and it has been widely used to model array-derived expression data in yeast and human [3,39]. The described SVD-based approach is effective for modelling both upregulated and downregulated genes, and the positive and negative values represent the upregulated and downregulated genes, respectively (Fig. 2A).

### Definition of promoter matrix

Prior to mathematical formulation, a promoter matrix *H*_*nxM *_was defined, where *n *was the number of the IL-1-responsive genes and *M *was the total number of TFBM candidates. The element *h*_*ij *_in *H*_*nxM *_represented the number of appearance of the *j-th *TFBM candidate on the 5'-end flanking region, 300 bp in length in the current study, of the *i-th *IL-1-responsive gene. The length of 300 bp was determined to minimize the least-square model error from the upstream regions of 100 bp to 5000 bp with a 100-bp interval. In this study, 512 TFBM candidates (*M *= 512), 5-bp DNA sequences including 5'-AAAAA-3', 5'-AAAAC-3', etc., were initially screened without considering polarity of DNA strands, and the critical TFBMs were selected by the SVD-based procedures described below. Since the length of motifs varies from 5 to 30 bp in TRANSFAC database, the 5-bp sequences were chosen as a potential core binding motif and their linkage to known motifs with redundancy was considered using TRANSFAC database.

### Formulation

Using the promoter matrix *H*_*nxm*_, the mRNA level of each IL-1-responsive gene was modelled [40]:

*z*= *H_*nxm*_x*    (1)

where *z* was the mRNA expression vector representing the logarithmic mRNA ratios for the 45 IL-1-responsive genes, and *x* was the state vector representing the role of TFBM candidates in achieving the observed values in *z*. Note that we used *M *as the total number of TFBM candidates (*M *= 512 in this study), *m *as the number of TFBMs in the SVD-based model, and $\hat{m}$ as the estimate of *m *based on Akaike information criterion below.

### Akaike information criterion

In order to avoid underfitting or overfitting the mRNA ratios with TFBM candidates, *AIC *was defined and used as an indicator of statistical measure [41]:

$AIC(m)=-2\log L(\underline{x},m)+2m\left(2\right)$

where *L*($\underline{\hat{x}}$ , *m*) was the likelihood function, and $\underline{\hat{x}}$ was the estimate of *x*. The value of $\underline{\hat{x}}$ was determined using the singular value decomposition procedure described below. The likelihood of the expression vector, *z*, with the estimate of the state vector, $\underline{\hat{x}}$ , was calculated:

$L(\underline{x},m)={\left(2\pi \sigma^{2}\right)}^{-\frac{n}{2}}\exp\{-\frac{1}{2}\sigma^{2}{\left(\underline{z}-H_{nxm}\underline{x}\right)}^{T}(\underline{z}-H_{nxm}\underline{x})\}\left(3\right)$

where *σ*^2 ^was a model error variance. Prior to constructing the final SVD-based model, a set of preliminary models for *m *= 1, 2, ..., *M *were built using the singular value decomposition procedure, and *AIC*(*m*) was minimized by treating *m *as a parameter. Note that *AIC*($\hat{m}$) ≤ *AIC*(*m*) for *m *= 1, 2, ..., *M*, and $\hat{m}$ = 8 in this study.

### Singular value decomposition (SVD)

SVD is a matrix decomposition technique which can be applied to any rectangular matrix. It decomposes a matrix into two orthogonal matrices and one eigenvalue matrix. Two orthogonal matrices represent the column and the row spaces in the original matrix, and the eigenvalue matrix relates these two spaces. In order to evaluate the contribution of 512 potential TFBMs to the IL-1 responses, the promoter matrix *H*_*nxM *_was factorized using SVD:

*H*_*nxM *_= *U*_*nxn*_Λ_*nxM*_*V*_*MxM*_^*T *^    (4)

where *U*_*nxn*_(*u*_1_, *u*_2_, ..., *u*_*n*_) was defined as the eigen gene matrix, Λ_*nxM*_(*λ*_1_, *λ*_2_, ..., *λ*_*n*_;*O*_*nXM*-*n*_) was a matrix containing *n *eigen values in the first n column vectors, and *V*_*MxM*_(*v*_1_, *v*_2_, ..., *v*_*M*_) was defined as an eigen TFBM matrix. Note that *U*_*nxn *_and *V*_*MxM *_are orthogonal and therefore *U*_*nxn*_^*T *^*U*_*nxn *_= *I*_*nxn *_and *V*_*MxM*_*V*_*MxM*_^*T *^= *I*_*MxM*_. In the *U*_*nxn *_space, the mRNA expression vector, *z*, can be expressed as a linear combination of the orthogonal vectors *u*_1_, *u*_2_,..., *u*_*n *_and the eigen values *λ*_1_, *λ*_2_,...,*λ*_*n *_with *k*_*i *_(*i *= 1, 2, ..., *n*):

$\underline{z}=\sum_{i=1}^{n}k_{i}\lambda_{i}{\underline{u}}_{i}\left(5\right)$

Determination of *k*_*i *_was achieved by projecting the vector *z* to *λ*_*i*_*u*_*i *_direction. Therefore, taking an inner product between *z* and *λ*_*i*_*u*_*i *_gave *k*_*i*_.

Since *u*_*i *_and *v*_*i *_are the associated bases in the gene space and the TFBM space respectively, the factor *k*_*i *_(*i *= 1, 2,..., *n*) for describing the expression in gene space can be used to model the contribution of individual TFBMs in the TFBM space. For instance, let us consider one extreme case where *z* was parallel to *u*_1_. Then, a contribution of TFBMs to *z* would be proportional to *v*_1 _and not affected by the other *v*_*i *_(*i *≠ 1) since the eigenvalue matrix Λ_nxM _does not have any non-diagonal components. Therefore, the elements in *v*_1 _would be used to indicate potential importance of M TFBM candidates. In a general case, the SVD procedure allowed us to evaluate n pairs of *u*_*i *_and *v*_*i *_through *λ*_*i *_and *k*_*i *_without conducting any combinatorial search. In order to model the gene space using the observed mRNA expression of *z*, the orthogonal vectors (*u*_1_, *u*_2_,..., *u*_*n*_) are linearly combined using *k*_*i *_(i = 1, 2,..., n). In order to model the TFBM space, a linear combination of the orthogonal vectors (*v*_1_, *v*_1_,..., *v*_*n*_) is made.

Based on the above rationale, we evaluated the linear combination of the eigen TFBM vectors in a form of $\underline{a}=\sum_{i=1}^{n}k_{i}{\underline{v}}_{i}$. This vector *a* plays the similar role of *z*in Eq. (5). M elements in *a *indicates the role and the contribution of M TFBM candidates. The positive/negative value suggests a stimulatory/inhibitory role, and a larger absolute value implies a stronger contribution. Therefore, the procedure to select $\hat{m}$ TFBMs is to choose a set of top $\hat{m}$ TFBMs whose value in *a *is larger than other (512 - $\hat{m}$) TFBMs. To include redundancy in TFBM consensus sequences, a weighted linear combination of elements in *a *can be used. In summary, the principal component analysis allows us to identify the principal expression components using the eigen gene vectors and to predict the principal TFBM using the eigen TFBM vectors. With the weighting factors defined from the observed value of *z*, the vector *a *indicates the predicted contribution of individual TFBM candidates to the observed expression pattern.

### Genetic Algorithm (GA) and Monte Carlo simulations

In order to evaluate the SVD-based prediction of TFBMs, the numerical simulations with GA were conducted using the procedure described previously [42]. In a chromosome-like bit map, 512 TFBM candidates were embedded:

*C *= [*c*_1_, *c*_2_,..., *c*_512_]     (6)

where each chromosomal element took "1" and "0" for inclusion and non-inclusion in the model, respectively. Note that $\sum_{i=1}^{512}c_{i}=\hat{m}$ for any chromosome, and the promoter matrix was constructed based on the value of each chromosomal element *c*_*i*_. Two hundred chromosomes represented the population, and one chromosome in the first generation corresponded to the SVD selection. In each generation, 100 chromosomes with smaller errors were recombined, and the other 100 chromosomes with larger errors were mutated. The model error was defined as ${\left|\underline{z}-H_{nxm}\hat{\underline{x}}\right|}^{2}$, and the state variable, *x*, was estimated using a least-square scheme:

$\hat{\underline{x}}={\left(H_{{nxm}^{T}}\;H_{nxm}\right)}^{-1}H_{{nxm}^{T}}\;\underline{z}\;\left(7\right)$

Note that *n *= 45 and *m *= $\hat{m}$ = 8 in GA. Monte Carlo simulation was also performed to evaluate numerically the SVD- and GA-based selection of TFBMs [42]. A set of $\hat{m}$ TFBMs was randomly chosen from 512 TFBM candidates, and the error distribution associated with the randomly selected TFBMs was compared to the error in the model-based prediction. The simulation was conducted 10,000 times.

### Linkage map among TFBMs

The 8 TFBM candidates, derived from the GA analysis, were linked to the biologically known TFBMs. We evaluated the 5-bp core consensus sequences identical to the known TFBMs using TRANSFAC database [43]. Since the motifs in the database ranges up to 30 bp, it is possible that a 5-bp TFBM candidate corresponds to multiple motifs in the database. Namely, the state vector could represent the combined role of binding motifs when the predicted motifs are shared among transcription factors.

## Supplementary Material

Additional File 1 • Part I – Experimental evaluation of the SVD-based model for IL1 responses. • Part II – SVD analysis for yeast Ras/cAMP signaling pathway. Click here for file

## References

[B1] de Jong H Modeling and simulation of genetic regulatory systems: a literature review J Comput Biol 2002 9 1 67 103 10.1089/10665270252833208 11911796

[B2] Lockhart DJ Winzeler EA Genomics, gene expression and DNA arrays Nature 2000 405 6788 827 836 10.1038/35015701 10866209

[B3] Bussemaker HJ Li H Siggia ED Regulatory element detection using correlation with expression Nat Genet 2001 27 2 167 171 10.1038/84792 11175784

[B4] Conlon EM Liu XS Lieb JD Liu JS Integrating regulatory motif discovery and genome-wide expression analysis Proc Natl Acad Sci U S A 2003 100 6 3339 3344 10.1073/pnas.0630591100 12626739 PMC152294

[B5] Lander ES Linton LM Birren B Nusbaum C Zody MC Baldwin J Devon K Dewar K Doyle M FitzHugh W Funke R Gage D Harris K Heaford A Howland J Kann L Lehoczky J LeVine R McEwan P McKernan K Meldrim J Mesirov JP Miranda C Morris W Naylor J Raymond C Rosetti M Santos R Sheridan A Sougnez C Stange-Thomann N Stojanovic N Subramanian A Wyman D Rogers J Sulston J Ainscough R Beck S Bentley D Burton J Clee C Carter N Coulson A Deadman R Deloukas P Dunham A Dunham I Durbin R French L Grafham D Gregory S Hubbard T Humphray S Hunt A Jones M Lloyd C McMurray A Matthews L Mercer S Milne S Mullikin JC Mungall A Plumb R Ross M Shownkeen R Sims S Waterston RH Wilson RK Hillier LW McPherson JD Marra MA Mardis ER Fulton LA Chinwalla AT Pepin KH Gish WR Chissoe SL Wendl MC Delehaunty KD Miner TL Delehaunty A Kramer JB Cook LL Fulton RS Johnson DL Minx PJ Clifton SW Hawkins T Branscomb E Predki P Richardson P Wenning S Slezak T Doggett N Cheng JF Olsen A Lucas S Elkin C Uberbacher E Frazier M Gibbs RA Muzny DM Scherer SE Bouck JB Sodergren EJ Worley KC Rives CM Gorrell JH Metzker ML Naylor SL Kucherlapati RS Nelson DL Weinstock GM Sakaki Y Fujiyama A Hattori M Yada T Toyoda A Itoh T Kawagoe C Watanabe H Totoki Y Taylor T Weissenbach J Heilig R Saurin W Artiguenave F Brottier P Bruls T Pelletier E Robert C Wincker P Smith DR Doucette-Stamm L Rubenfield M Weinstock K Lee HM Dubois J Rosenthal A Platzer M Nyakatura G Taudien S Rump A Yang H Yu J Wang J Huang G Gu J Hood L Rowen L Madan A Qin S Davis RW Federspiel NA Abola AP Proctor MJ Myers RM Schmutz J Dickson M Grimwood J Cox DR Olson MV Kaul R Shimizu N Kawasaki K Minoshima S Evans GA Athanasiou M Schultz R Roe BA Chen F Pan H Ramser J Lehrach H Reinhardt R McCombie WR de la Bastide M Dedhia N Blocker H Hornischer K Nordsiek G Agarwala R Aravind L Bailey JA Bateman A Batzoglou S Birney E Bork P Brown DG Burge CB Cerutti L Chen HC Church D Clamp M Copley RR Doerks T Eddy SR Eichler EE Furey TS Galagan J Gilbert JG Harmon C Hayashizaki Y Haussler D Hermjakob H Hokamp K Jang W Johnson LS Jones TA Kasif S Kaspryzk A Kennedy S Kent WJ Kitts P Koonin EV Korf I Kulp D Lancet D Lowe TM McLysaght A Mikkelsen T Moran JV Mulder N Pollara VJ Ponting CP Schuler G Schultz J Slater G Smit AF Stupka E Szustakowski J Thierry-Mieg D Thierry-Mieg J Wagner L Wallis J Wheeler R Williams A Wolf YI Wolfe KH Yang SP Yeh RF Collins F Guyer MS Peterson J Felsenfeld A Wetterstrand KA Patrinos A Morgan MJ Szustakowki J de Jong P Catanese JJ Osoegawa K Shizuya H Choi S Chen YJ Initial sequencing and analysis of the human genome Nature 2001 409 6822 860 921 10.1038/35057062 11237011

[B6] Thompson W Palumbo MJ Wasserman WW Liu JS Lawrence CE Decoding human regulatory circuits Genome Res 2004 14 10A 1967 1974 10.1101/gr.2589004 15466295 PMC524421

[B7] Karolchik D Baertsch R Diekhans M Furey TS Hinrichs A Lu YT Roskin KM Schwartz M Sugnet CW Thomas DJ Weber RJ Haussler D Kent WJ The UCSC Genome Browser Database Nucleic Acids Res 2003 31 1 51 54 10.1093/nar/gkg129 12519945 PMC165576

[B8] Gupta M Liu JS Discovery of Conserved Sequence Patterns Using a Stochastic Dictionary Model Journal of the American Statistical Association 2003 461 55-66 98

[B9] Grad YH Roth FP Halfon MS Church GM Prediction of similarly acting cis-regulatory modules by subsequence profiling and comparative genomics in Drosophila melanogaster and D.pseudoobscura Bioinformatics 2004 20 16 2738 2750 10.1093/bioinformatics/bth320 15145800

[B10] Sharan R Ovcharenko I Ben-Hur A Karp RM CREME: a framework for identifying cis-regulatory modules in human-mouse conserved segments Bioinformatics 2003 19 Suppl 1 i283 91 10.1093/bioinformatics/btg1039 12855471

[B11] Keles S van der Laan M Eisen MB Identification of regulatory elements using a feature selection method Bioinformatics 2002 18 9 1167 1175 10.1093/bioinformatics/18.9.1167 12217908

[B12] Xu Y Selaru FM Yin J Zou TT Shustova V Mori Y Sato F Liu TC Olaru A Wang S Kimos MC Perry K Desai K Greenwald BD Krasna MJ Shibata D Abraham JM Meltzer SJ Artificial neural networks and gene filtering distinguish between global gene expression profiles of Barrett's esophagus and esophageal cancer Cancer Res 2002 62 12 3493 3497 12067993

[B13] Vincenti MP Brinckerhoff CE Early response genes induced in chondrocytes stimulated with the inflammatory cytokine interleukin-1beta Arthritis Res 2001 3 6 381 388 10.1186/ar331 11714393 PMC64850

[B14] Heller RA Schena M Chai A Shalon D Bedilion T Gilmore J Woolley DE Davis RW Discovery and analysis of inflammatory disease-related genes using cDNA microarrays Proc Natl Acad Sci U S A 1997 94 6 2150 2155 10.1073/pnas.94.6.2150 9122163 PMC20056

[B15] Elliott SF Coon CI Hays E Stadheim TA Vincenti MP Bcl-3 is an interleukin-1-responsive gene in chondrocytes and synovial fibroblasts that activates transcription of the matrix metalloproteinase 1 gene Arthritis Rheum 2002 46 12 3230 3239 10.1002/art.10675 12483727

[B16] Chadjichristos C Ghayor C Kypriotou M Martin G Renard E Ala-Kokko L Suske G de Crombrugghe B Pujol JP Galera P Sp1 and Sp3 transcription factors mediate interleukin-1 beta down-regulation of human type II collagen gene expression in articular chondrocytes J Biol Chem 2003 278 41 39762 39772 10.1074/jbc.M303541200 12888570

[B17] Francois M Richette P Tsagris L Raymondjean M Fulchignoni-Lataud MC Forest C Savouret JF Corvol MT Peroxisome proliferator-activated receptor-gamma down-regulates chondrocyte matrix metalloproteinase-1 via a novel composite element J Biol Chem 2004 279 27 28411 28418 10.1074/jbc.M312708200 15090544

[B18] Imamura T Imamura C Iwamoto Y Sandell LJ Transcriptional Co-activators CREB-binding protein/p300 increase chondrocyte Cd-rap gene expression by multiple mechanisms including sequestration of the repressor CCAAT/enhancer-binding protein J Biol Chem 2005 280 17 16625 16634 10.1074/jbc.M411469200 15722556

[B19] Gardner TS di Bernardo D Lorenz D Collins JJ Inferring genetic networks and identifying compound mode of action via expression profiling Science 2003 301 5629 102 105 10.1126/science.1081900 12843395

[B20] Troyanskaya O Cantor M Sherlock G Brown P Hastie T Tibshirani R Botstein D Altman RB Missing value estimation methods for DNA microarrays Bioinformatics 2001 17 6 520 525 10.1093/bioinformatics/17.6.520 11395428

[B21] Chuang HYH Chen L Efficient Computation of the Singlular Value Decomposition on Cube Connected SIMD Machine: Reno. 1989 276 282

[B22] Alter O Brown PO Botstein D Singular value decomposition for genome-wide expression data processing and modeling Proc Natl Acad Sci U S A 2000 97 18 10101 10106 10.1073/pnas.97.18.10101 10963673 PMC27718

[B23] Liu Y Sun HB Yokota H Regulating gene expression using optimal control theory Proc 3rd IEEE Sym Bioinfo Bioeng 2003 1 3

[B24] Holter NS Mitra M Maritan A Cieplak M Banavar JR Fedoroff NV Fundamental patterns underlying gene expression profiles: simplicity from complexity Proc Natl Acad Sci U S A 2000 97 15 8409 8414 10.1073/pnas.150242097 10890920 PMC26961

[B25] Holland JH Adaptation in natural and artificial systems 1975 Ann Arbor , The University of Michigan Press

[B26] Li L Weinberg CR Darden TA Pedersen LG Gene selection for sample classification based on gene expression data: study of sensitivity to choice of parameters of the GA/KNN method Bioinformatics 2001 17 12 1131 1142 10.1093/bioinformatics/17.12.1131 11751221

[B27] Grether-Beck S Buettner R Krutmann J Ultraviolet A radiation-induced expression of human genes: molecular and photobiological mechanisms Biol Chem 1997 378 11 1231 1236 9426182

[B28] Eastman Q Grosschedl R Regulation of LEF-1/TCF transcription factors by Wnt and other signals Curr Opin Cell Biol 1999 11 2 233 240 10.1016/S0955-0674(99)80031-3 10209158

[B29] Tan L Peng H Osaki M Choy BK Auron PE Sandell LJ Goldring MB Egr-1 mediates transcriptional repression of COL2A1 promoter activity by interleukin-1beta J Biol Chem 2003 10.1074/jbc.M301676200 12637574

[B30] Philipsen S Suske G A tale of three fingers: the family of mammalian Sp/XKLF transcription factors Nucleic Acids Res 1999 27 15 2991 3000 10.1093/nar/27.15.2991 10454592 PMC148522

[B31] Vincenti MP Coon CI Brinckerhoff CE Nuclear factor kappaB/p50 activates an element in the distal matrix metalloproteinase 1 promoter in interleukin-1beta-stimulated synovial fibroblasts Arthritis Rheum 1998 41 11 1987 1994 10.1002/1529-0131(199811)41:11<1987::AID-ART14>3.0.CO;2-8 9811054

[B32] Ding GJ Fischer PA Boltz RC Schmidt JA Colaianne JJ Gough A Rubin RA Miller DK Characterization and quantitation of NF-kappaB nuclear translocation induced by interleukin-1 and tumor necrosis factor-alpha. Development and use of a high capacity fluorescence cytometric system J Biol Chem 1998 273 44 28897 28905 10.1074/jbc.273.44.28897 9786892

[B33] Barnes PJ Karin M Nuclear factor-kappaB: a pivotal transcription factor in chronic inflammatory diseases N Engl J Med 1997 336 15 1066 1071 10.1056/NEJM199704103361506 9091804

[B34] Sun HB Malacinski GM Yokota H Promoter competition assay for analyzing gene regulation in joint tissue engineering Front Biosci 2002 7 a169 74 12133815 10.2741/a751

[B35] Sun HB Liu Y Qian L Yokota H Model-based analysis of matrix metalloproteinase expression under mechanical shear Ann Biomed Eng 2003 31 2 171 180 10.1114/1.1540635 12627825

[B36] Collins FS Green ED Guttmacher AE Guyer MS A vision for the future of genomics research Nature 2003 422 6934 835 847 10.1038/nature01626 12695777

[B37] Venter JC Adams MD Myers EW Li PW Mural RJ Sutton GG Smith HO Yandell M Evans CA Holt RA Gocayne JD Amanatides P Ballew RM Huson DH Wortman JR Zhang Q Kodira CD Zheng XH Chen L Skupski M Subramanian G Thomas PD Zhang J Gabor Miklos GL Nelson C Broder S Clark AG Nadeau J McKusick VA Zinder N Levine AJ Roberts RJ Simon M Slayman C Hunkapiller M Bolanos R Delcher A Dew I Fasulo D Flanigan M Florea L Halpern A Hannenhalli S Kravitz S Levy S Mobarry C Reinert K Remington K Abu-Threideh J Beasley E Biddick K Bonazzi V Brandon R Cargill M Chandramouliswaran I Charlab R Chaturvedi K Deng Z Di Francesco V Dunn P Eilbeck K Evangelista C Gabrielian AE Gan W Ge W Gong F Gu Z Guan P Heiman TJ Higgins ME Ji RR Ke Z Ketchum KA Lai Z Lei Y Li Z Li J Liang Y Lin X Lu F Merkulov GV Milshina N Moore HM Naik AK Narayan VA Neelam B Nusskern D Rusch DB Salzberg S Shao W Shue B Sun J Wang Z Wang A Wang X Wang J Wei M Wides R Xiao C Yan C Yao A Ye J Zhan M Zhang W Zhang H Zhao Q Zheng L Zhong F Zhong W Zhu S Zhao S Gilbert D Baumhueter S Spier G Carter C Cravchik A Woodage T Ali F An H Awe A Baldwin D Baden H Barnstead M Barrow I Beeson K Busam D Carver A Center A Cheng ML Curry L Danaher S Davenport L Desilets R Dietz S Dodson K Doup L Ferriera S Garg N Gluecksmann A Hart B Haynes J Haynes C Heiner C Hladun S Hostin D Houck J Howland T Ibegwam C Johnson J Kalush F Kline L Koduru S Love A Mann F May D McCawley S McIntosh T McMullen I Moy M Moy L Murphy B Nelson K Pfannkoch C Pratts E Puri V Qureshi H Reardon M Rodriguez R Rogers YH Romblad D Ruhfel B Scott R Sitter C Smallwood M Stewart E Strong R Suh E Thomas R Tint NN Tse S Vech C Wang G Wetter J Williams S Williams M Windsor S Winn-Deen E Wolfe K Zaveri J Zaveri K Abril JF Guigo R Campbell MJ Sjolander KV Karlak B Kejariwal A Mi H Lazareva B Hatton T Narechania A Diemer K Muruganujan A Guo N Sato S Bafna V Istrail S Lippert R Schwartz R Walenz B Yooseph S Allen D Basu A Baxendale J Blick L Caminha M Carnes-Stine J Caulk P Chiang YH Coyne M Dahlke C Mays A Dombroski M Donnelly M Ely D Esparham S Fosler C Gire H Glanowski S Glasser K Glodek A Gorokhov M Graham K Gropman B Harris M Heil J Henderson S Hoover J Jennings D Jordan C Jordan J Kasha J Kagan L Kraft C Levitsky A Lewis M Liu X Lopez J Ma D Majoros W McDaniel J Murphy S Newman M Nguyen T Nguyen N Nodell M Pan S Peck J Peterson M Rowe W Sanders R Scott J Simpson M Smith T Sprague A Stockwell T Turner R Venter E Wang M Wen M Wu D Wu M Xia A Zandieh A Zhu X The sequence of the human genome Science 2001 291 5507 1304 1351 10.1126/science.1058040 11181995

[B38] Davuluri RV Grosse I Zhang MQ Computational identification of promoters and first exons in the human genome Nat Genet 2001 29 4 412 417 10.1038/ng780 11726928

[B39] Liu Y Yokota H Modelling and idenification of transcription-factor binding motifs in human chondrogenesis Systems Biology 2004 1 1 85 92 10.1049/sb:20045012 17052118

[B40] Qian L Liu Y Sun HB Yokota H Systems analysis of matrix metalloproteinase mRNA expression in skeletal tissues Front Biosci 2002 7 a126 34 12045007 10.2741/qian

[B41] Akaike H A new look at the statistical model identification IEEE Transactions on Automatic Control 1974 AC-19 716 723 10.1109/TAC.1974.1100705

[B42] Liu Y Yokota H Modelling and identification of transcription-factor binding motifs in human chondrogenesis Systems Biology 2004 1 1 85 92 10.1049/sb:20045012 17052118

[B43] Wingender E Dietze P Karas H Knuppel R TRANSFAC: a database on transcription factors and their DNA binding sites Nucleic Acids Res 1996 24 1 238 241 10.1093/nar/24.1.238 8594589 PMC145586

