# Supplementary material for: Principal component analysis for predicting transcription-factor binding motifs from array-derived data
Source: BMC Bioinformatics. 2005 Nov 18;6:276. doi: 10.1186/1471-2105-6-276 (PMC1316881; doi:10.1186/1471-2105-6-276)
Supplement: Additional File 1 — • Part I – Experimental evaluation of the SVD-based model for IL1 responses. • Part II – SVD analysis for yeast Ras/cAMP signaling pathway. [file 1471-2105-6-276-S1.doc]

**Additional File**

Summary: Two parts included here are:

- Part I - Experimental evaluation of the SVD-based model for IL1 responses
- Part II - SVD analysis for yeast Ras/cAMP signaling pathway

**Part I. Experimental evaluation of the SVD-based model for IL1 responses**

In order to evaluate the SVD-based model for the IL1 responses, we conducted a gel shift assay and a gene reporter assay using C28/I-2 human chondrocytes. The SVD and GA analysis both predicted the stimulatory role of 5’-CAGGC-3’, and therefore we focused on this *de novo* TFBM candidate. The results clearly showed its stimulatory effects in response to IL1.

First, in the gel shift assay incubation with the nuclear extracts isolated from the IL-1β-treated cells retarded a mobility of the DNA fragments containing 5’-CAGGC-3’ (Fig. S1A). A radioactive intensity of the two shifted bands was reduced by the cold competitor specific to the DNA fragments but not by the nonspecific competitor. Second, the reporter gene assay revealed that the 5’-CAGGC-3’ sequence elevated induction of the reporter gene by 22.1% in the presence of 5 ng/ml IL-1β (Fig. S1B). The NFκB construct, used as a positive control, increased IL-1β-driven induction by 35.0%.

Submission of these results to molecular biology journal is in preparation.

| 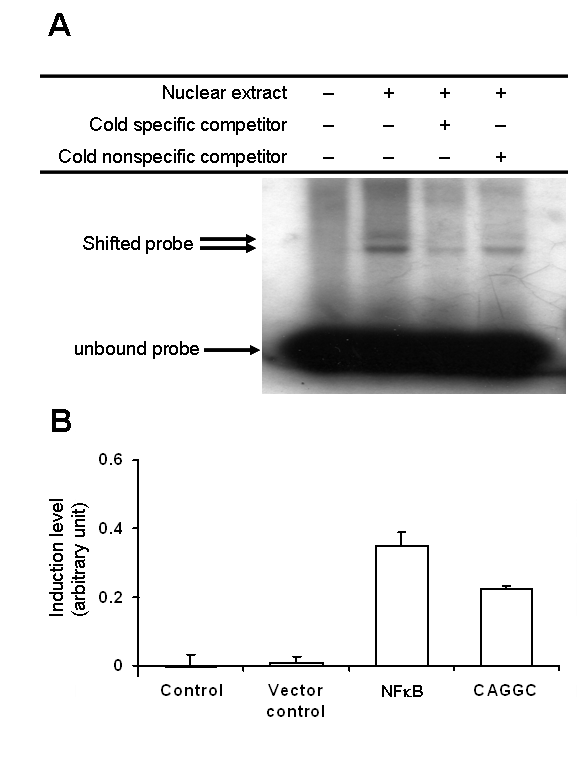 | Figure S1. Gel shift assay and reporter gene assay. (**A**) Gel shift assay for a putative TFBM, 5’-CAGGC-3’. Four lanes correspond to (i) negative control lane; (ii) experimental lane with no specific/nonspecific competitors; (iii) control lane with specific competitors; and (iv) control lane with nonspecific competitors. The bands in the third lane are suppressed because of the specific competitors. Two bands in the second lane suggest two complexes with differential gel mobility. (**B**) Reporter gene assay for 5’-CAGGC-3’. The assay was conducted using the SEAP Reporter System 3. Four copies of the selected 5-bp DNA sequences were inserted into pTAL-SEAP vector (4.8 kb). The vector without an insert was used as negative control, and the vector with 4 copies of the NFκB binding site was used as positive control. The plasmids were transfected into using the Effectene Transfection Reagent. Cells were incubated with IL-1β for 6 h before the culture medium was harvested for the SEAP activity assay. Induction of the reporter gene was determined by measuring MUP fluorescence at 360/449 nm (excitation/emission) with a FluoroMax-2 spectrofluorometer (Instruments SA Inc.). |
| --- | --- |

**Part II. SVD analysis for yeast Ras/cAMP signaling pathway**

Yeast Ras/cAMP signaling pathway: Ras/cAMP is a key regulator of cell growth and stress responses in all eukaryotic cells. Genetic, biochemical, and molecular studies in *S. cerevisae*, *C.elegans* and human have positioned Ras/cAMP centrally in signal transduction pathways that respond to diverse extracellular stimuli including cytokines and mechanical stress. We used the yeast expression data on the Ras/cAMP signaling pathway that are publicly available from NCBI gene expression omnibuss (accession numbers GSM9142, and GSM9174-9182). Two *S. cerevisiae* strains, FY23 wild-type and PDE2 mutant, were used in the study. The mutant exhibits a constitutive activation of the Ras/cAMP pathway, and comparison of their expression profiles reveals transcriptional regulation medicated by the Ras/cAMP signaling pathway. The advantage of using the yeast gene expression data are as follows: First, Ras/cAMP is a well conserved signaling pathway in eukaryotes and suitable for functional phylogenetic footprinting. Second, many transcription factors activated in the pathway are known in yeast (e.g., ADR1, MSN2, SWI4, and HAC1) and human (e.g., NFκB, Elk1, SRF, and ATF2), and the prediction can be evaluated based on the known factors. Particularly, the CRE motif ([A/G][A/C][T/C]GCAGT) is conserved as a cAMP responsive element in yeast and human. Third, multiple known and potentially de novo binding motifs are influenced by Ras/cAMP and therefore the expression data allow us to test the ability of searching a combination of binding motifs with the proposed algorithm.

Genes in the model: We included the genes whose expression levels were altered through Ras/cAMP pathway. Sixty-one genes were selected from tables 6, 7, 8 and 9 from Jones et al. (*Physiol. Genomics* 16:107-118, 2003). Their Gene ID, differential expression levels between mutant and control, and their functions were listed in Table S1.

Table S1. Summary of genes included in the model

| Gene ID | Fold change | Gene Function |
| --- | --- | --- |
| PGM2 | -1.779 | Phosphoglucomutase, major isoform |
| TSL1 | -1.572 | alpha, alpha-Trehalose-phosphate synthase, 123-kDa subunit |
| GPH1 | -1.535 | Glycogen phosphorylase |
| GAC1 | -1.475 | SerThr phosphoprotein phosphatase 1, regulatory chain |
| ADR1 | -1.463 | Zinc-finger transcription factor |
| YOR1 | -1.454 | ATP-binding cassette transporter protein |
| MSN2 | -1.420 | Stress-responsive regulatory protein |
| DDR48 | -1.405 | Heat-shock protein |
| TPS2 | -1.402 | alpha, alpha-Trehalose-phosphate synthase, 102-kDa subunit |
| SWI4 | -1.402 | Transcription factor |
| SIP4 | -1.401 | Interacts with SNF1 protein kinase |
| UBA1 | -1.392 | E1-like (ubiquitin-activating) enzyme |
| CTT1 | -1.391 | Catalase T, cytosolic |
| MPT5 | -1.386 | Multicopy suppressor of POP2 |
| HAC1 | -1.369 | Transcription factor |
| CAT8 | -1.364 | Transcription factor involved in gluconeogenesis |
| MIG2 | -1.362 | C2H2 zinc-finger protein |
| RAD5 | -1.353 | DNA helicase |
| PDR5 | -1.349 | Pleiotropic drug resistance protein |
| YOR273C | -1.343 | Similarity to resistance proteins |
| CHA4 | -1.335 | Transcription factor |
| YCF1 | -1.333 | Glutathione S-conjugate transporter, vacuolar |
| HSP78 | -1.301 | Heat-shock protein of clpb family of ATP-dependent proteases |
| TPO1 | -1.297 | Similarity to multidrug resistance proteins |
| UBI4 | 1.290 | Ubiquitin |
| YDL100C | 1.300 | Similarity to *E.coli* aresenical pump-driving APTase |
| GRX3 | 1.301 | Member of the subfamily of yeast glutaredoxins (Grx3, Grx4, and Grx5) |
| UBC4 | 1.302 | E2 ubiquitin-conjugating enzyme |
| YHP1 | 1.302 | Strong similarity to Yox1p |
| ALK1 | 1.305 | DNA damage-responsive protein |
| PAU6 | 1.310 | Strong similarity to members of the Tir1p/Tip1p family |
| TRX1 | 1.311 | Thioredoxin I |
| GLR1 | 1.337 | Glutathione reductase (NADPH) |
| TEC1 | 1.342 | Ty transcription activator |
| TIP1 | 1.347 | Esterase |
| CYC7 | 1.349 | Cytochrome-c isoform 2 |
| TRX2 | 1.352 | Thioredoxin II |
| DDP1 | 1.358 | Diadenosine hexaphosphate (Ap6A) hydrolase |
| LEU3 | 1.380 | Transcription factor |
| RIM1 | 1.389 | ssDNA-binding protein, mitochondrial |
| SRL1 | 1.406 | Similarity to vanadate sensitive suppresor Svs1p |
| GRX4 | 1.415 | Member of the subfamily of yeast glutaredoxins (Grx3, Grx4, and Grx5) |
| SPS18 | 1.423 | Sporulation-specific zinc-fingure protein |
| MAL33 | 1.447 | Maltose fermentation regulatory protein |
| CUP5 | 1.462 | H+-ATPase V0 domain 17kDa subunit, vacuolar |
| MRPL25 | 1.476 | Ribosomal protein YmL25, mitochondrial |
| SSU72 | 1.487 | Suppressor of cs mutant of sua7 |
| RPL18A | 1.496 | 60S Large subunit ribosomal protein S18.e |
| RPS14A | 1.504 | 40S Ribosomal protein S14.e |
| RPS10A | 1.512 | Ribosomal protein S10.e |
| RPS3 | 1.517 | Ribosomal protein S3.e |
| RPS2 | 1.536 | 40S Small subunit ribosomal protein |
| MRPL40 | 1.541 | Ribosomal protein of the large subunit (YmL40), mitochondrial |
| RPL19B | 1.548 | 60S Large subunit ribosomal protein L19.e |
| GCN4 | 1.561 | Transcriptional activator of amino acid biosynthetic genes |
| RPS5 | 1.566 | Ribosomal protein S5.e |
| YJL206C | 1.574 | Similarity to YIL130p and Put3p |
| YPR015C | 1.586 | Similarity to transcription factors |
| RSM19 | 1.601 | Strong similarity to Mycoplasma ribosomal protein S19 |
| MPH1 | 1.996 | Protection of the genome from spontaneous and chemically induced damage |
| FKH1 | 2.789 | Similarity to Drosophila fork head protein |

Akaike information criterion (AIC): In order to estimate the proper number of TFBMs in the model, AIC was calculated (Fig. S3). The minimum AIC was obtained with 10 TFBMs ($\hat{m}$=10).

| 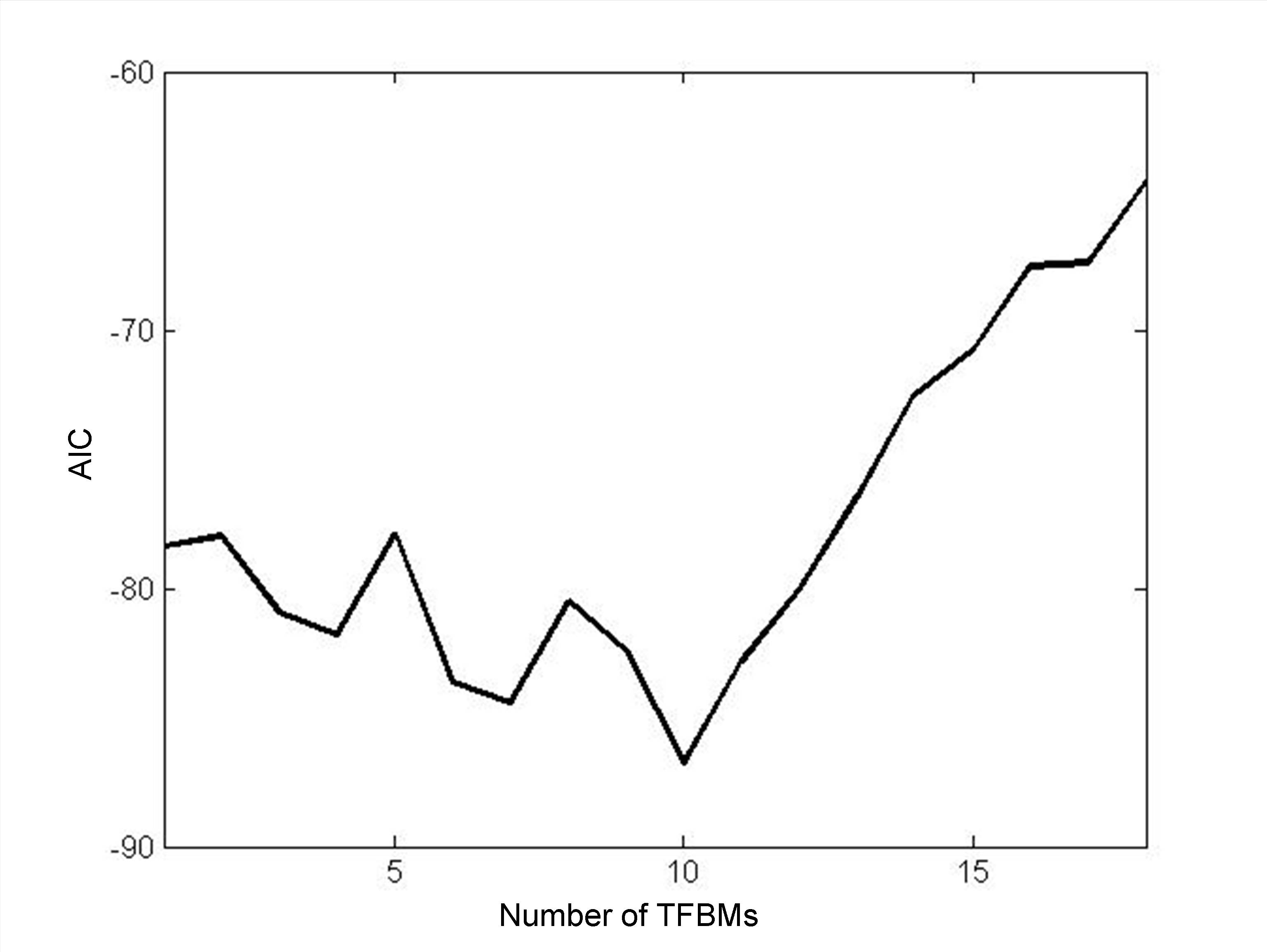 | Figure S3. Selection of number of TFBMs. The figure shows AIC as a function of *m* (number of TFBMs). The minimum AIC value, $\hat{m}$, was determined as 10. |
| --- | --- |

Singular value decomposition: We built the promoter matrix using 500-bp upstream flanking sequences, and decomposed it into three matrices such as *U*, *Λ* and *V*. Note that in this yeast study *n* = 61 (number of genes), *M* = 512 (TFBM candidates in total), and $\hat{m}$ = 10 (number of TFBMs in the SVD-based model). Out of 61 eigen values, the primary and secondary eigen values were 207.08 and 41.61.

In Figs. S4 and S5, we illustrate the eigen gene matrix U (Fig. S4A), eigen values in Λ (Fig. S4B), weighting factors *k_i_* (Fig. S4C), eigen TFBM matrix V (Fig. S5A), weighted eigen TFBM vectors (Fig. S5B), and the values indicating a contribution factor of TFBM candidates (Fig. S5C). The format is identical to the original manuscript on IL1 responses.

| 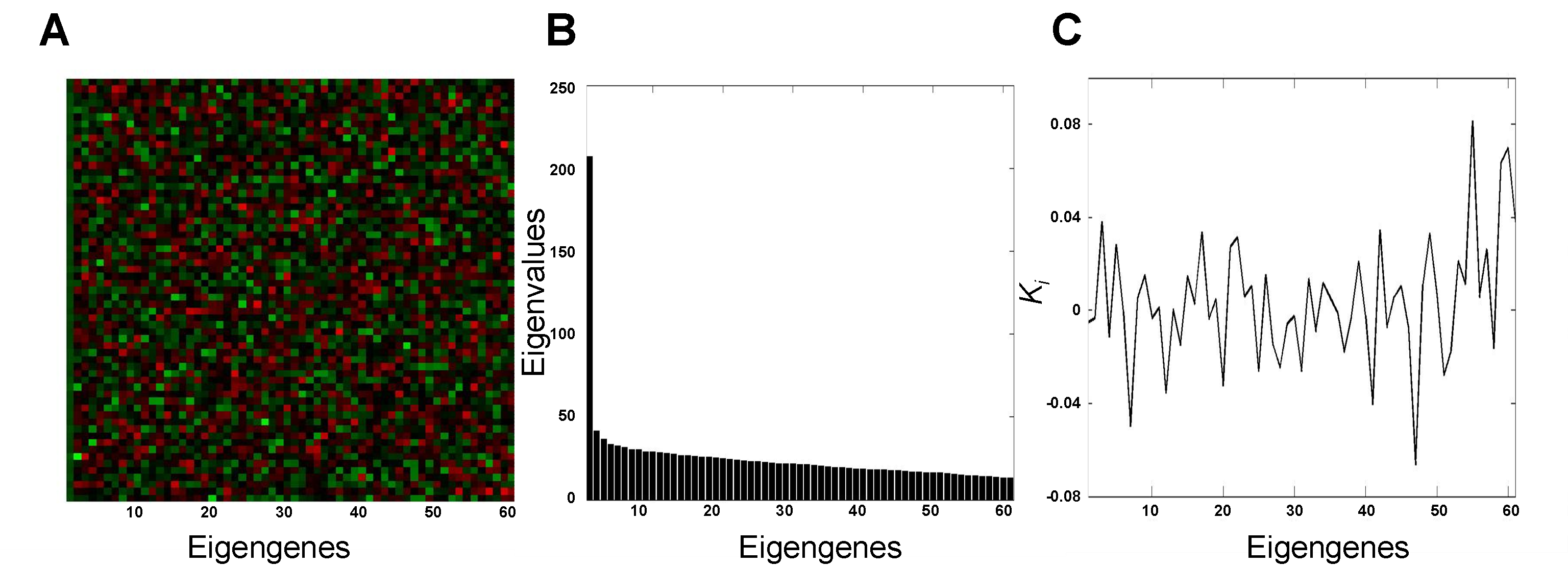 |
| --- |
| Figure S4. SVD analysis for the 61 Ras/cAMP-regulated genes. (A) Sixty-one eigen genes in the matrix *U* in *H = UΛV^T^*. (B) Eigen values, *λ_1_, λ_2_, …, λ_61_*, in the matrix *Λ*. (C) Weighting factors, *k_i_*, for the *i-th* eigen gene. |

| 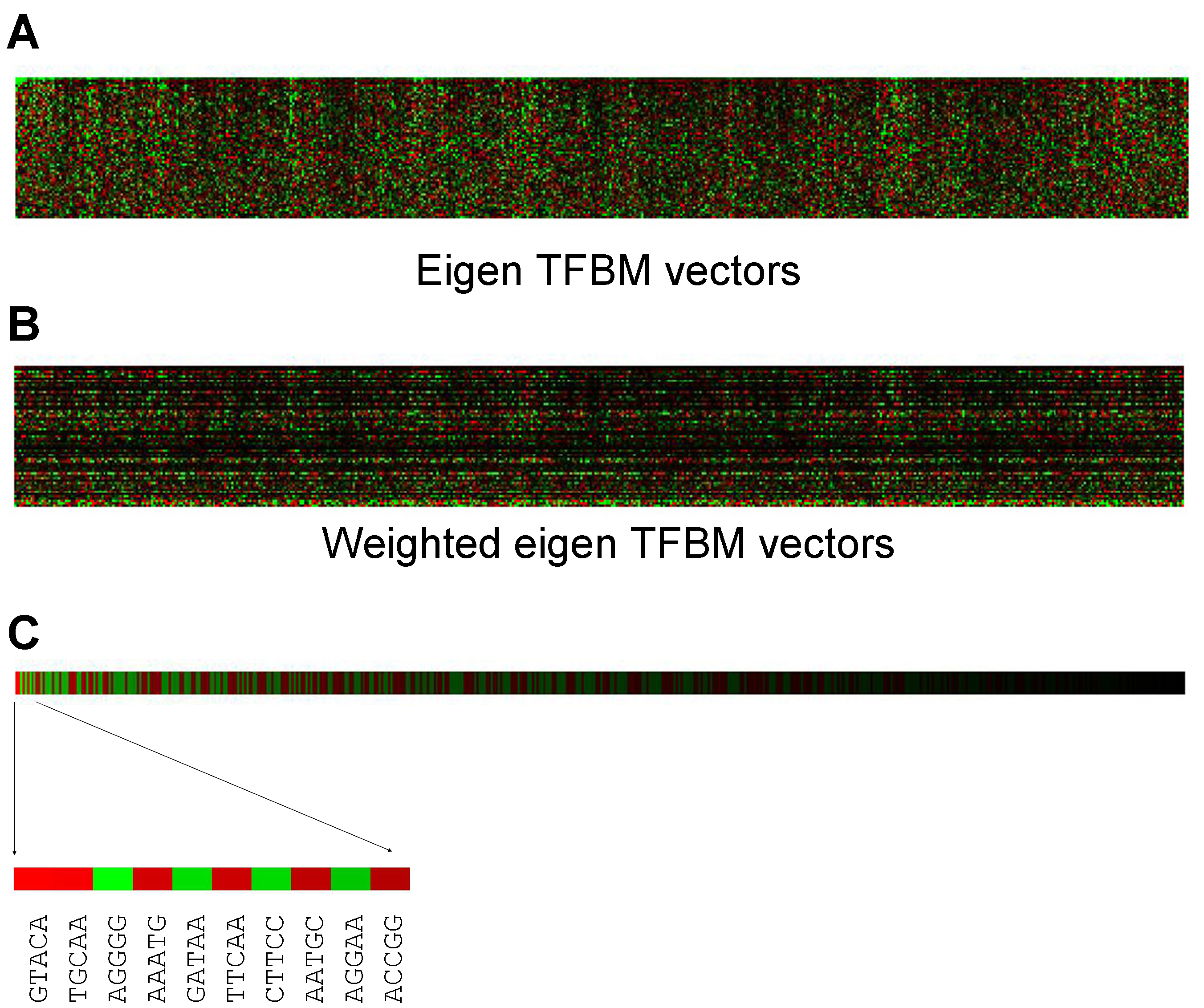 | Figure S5. SVD-based selection of TFBMs. (A) Eigen TFBM vectors in the matrix *V^T^* in *H = UΛV^T^*. (B) Weighted eigen TFBM vectors with the weighting factor, *k_i_*. (C) Putative TFBMs predicted from the SVD analysis. |
| --- | --- |

Monte-Carlo simulation: Monte-Carlo simulation was conducted to evaluate the SVD-based selections of TFBMs. The sum square error for the SVD-based model (7.63) was significantly lower than those based on random selection of TFBMs (14.68 ± 1.10, *N* = 10,000) (Figure S6).

| 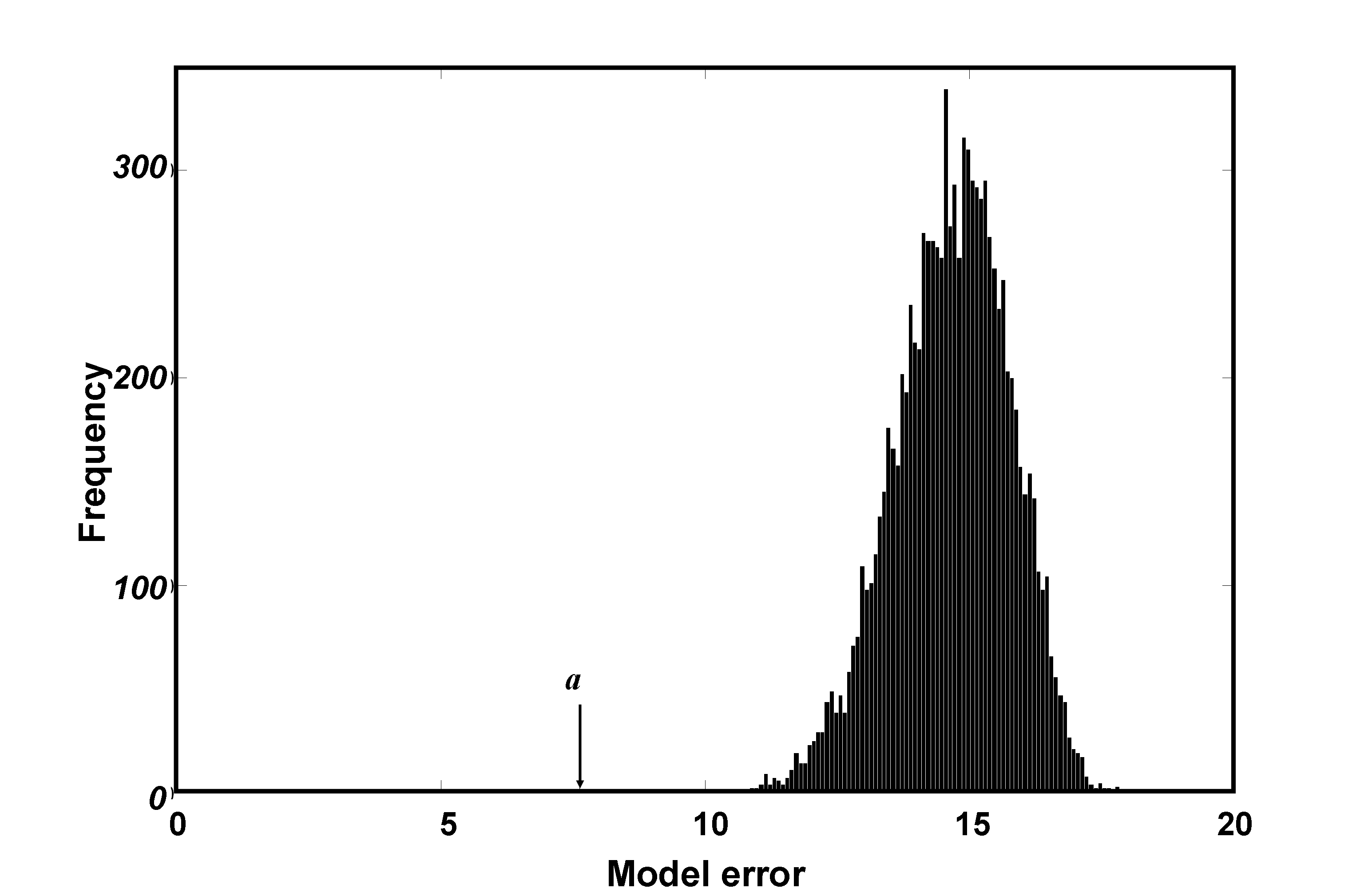 | Figure S6. Model error in Monte-Carlo simulation. The label, *a,* indicates the model error of the SVD-based model. |
| --- | --- |

Correspondences with known TRANSFAC database: The SVD procedure predicted 10 TFBM candidates including: 5’-GTACA-3', 5’-TGCAA-3', 5’-AGGGG-3', 5’-AAATG-3', 5’-GATAA-3', 5’-TTCAA-3', 5’-CTTCC-3', 5’-AATGC-3', 5’-AGGAA-3', and 5’-ACCGG-3'. In order to evaluate their biological relevance, sequence similarities with known TFBMs in TRANSFAC databases were examined (Table S2). Note that 5’-AGGGG-3’ and 5’-AATGC-3’, predicted in the SVD-based model, are part of a consensus sequence of STRE (stress-response element) and CRE (cAMP responsive element) respectively, which are known to play a major role in Ras/cAMP signaling pathway.

Table S2. Linkage between the predicted TFBMs and

the biologically known TFBMs in TRANSFAC database

| **Predicted TFBMs** | **Known TFBMs in TRANSFAC** | **Consensus sequences** | **Descriptions about transcription factors** |
| --- | --- | --- | --- |
| GTACA | bZIP911 | GRTGACG**TGTAC** | bZIP transcription factor from Antirrhinum majus |
|  | AR | G**GTACA**NNRTGTTCT | androgen receptor |
| TGCAA | Oct-1 | TA**TGCAA**ATN | Octamer binding factor 1 |
|  | CHOP:C/EBPα | NNR**TGCAA**TMCCC | heterodimers of CHOP and C/EBPα |
| AGGGG | STRE | TM**AGGGG**N | stress-response element |
|  | STRE | M**AGGGG**N | MSN2/MSN4, STRE (stress response element), S. cerevisiae |
|  | MZF1 | KNNNK**AGGGG**NAA | MZF1 |
|  | PPARα:RXR-α | NNRGGTCAT**WGGGG**TSANG | PPAR-α:RXR-α heterodimer |
| AAATG | STAT3 | ATTTCCSGG**AARTG** | signal transducer and activator of transcription 3 |
|  | MADS-A | ADWCCAA**AAATG**GAAA | determines identity of floral meristem and sepal development |
| GATAA | Evi-1 | A**GATAA**GATAA | ectopic viral integration site 1 encoded factor |
|  | GATA-x | **GATAA**GNM | GATA-binding factor 1 |
|  | mtTFA | KNC**TTATC** | mitochondrial transcription factor A |
| TTCAA | GCNF | TCAAG**KTCAA**GKTCA | GCNF (germ cell nuclear factor) |
| CTTCC | Ets | A**CTTCC**TS |  |
|  | GABP | VCC**GGAAG**NGCR | GA binding protein |
|  | ETS | ANNCA**CTTCC**TG |  |
|  | NRF-2 | ACC**GGAAG**NG | nuclear respiratory factor 2 |
|  | NERF1a | YRNCA**GGAAG**YRNSTBDS | new ets-related factor 1a |
|  | E74A | AAYCC**GGAAG**T | E74A |
| AATGC | CRE | **RMYGC**AGT | cAMP responsive element |
|  | AbaA | YNNBYY**NCATT**CCNNNNNN | AbaA |
|  | POU3F2 | AT**GMATW**WATTCAT |  |
| AGGAA | ETS | CAC**TTCCT**G |  |
|  | Helios A | WNW**AGGAA**AAN |  |
|  | PU.1 | WG**AGGAA**G |  |
|  | STAT6 | NNY**TTCCY** | signal transducer and activator of transcription 6 |
| ACCGG | LEU3 | HGCCGGT**ACCGG**YB | LEU3, S. cerevisiae |
|  | NRF-2 | **ACCGG**AAGNG | nuclear respiratory factor 2 |

Nucleotides in the consensus sequences are represented using IUB code: A, C, G, T, R = AG, Y = CT, K = GT, M = AC, S = GC, W = AT, B = CGT, D = AGT, H = ACT, V = ACG, N = ACGT.
